# Elevated hydrostatic pressure destabilizes VE-cadherin junctions in a time and shear stress dependent manner: An endothelium-on-chip study

**DOI:** 10.1063/5.0275985

**Published:** 2025-08-20

**Authors:** Pranav Vasanthi Bathrinarayanan, Thomas Abadie, Patricia Perez Esteban, D. Vigolo, M. J. H. Simmons, L. M. Grover

**Affiliations:** 1 School of Chemical Engineering, University of Birmingham, Edgbaston, Birmingham B15 2TT, United Kingdom; 2 Healthcare Technologies Institute, School of Chemical Engineering, University of Birmingham, Edgbaston, Birmingham B15 2TT, United Kingdom; 3 School of Biomedical Engineering, The University of Sydney, Sydney, New South Wales 2006, Australia; 4 The University of Sydney Nano Institute, The University of Sydney, Sydney, New South Wales 2006, Australia

## Abstract

Despite the effects of shear stress on endothelial biology having been extensively researched, the effects of hydrostatic vascular pressure at extremely low shear stresses have been largely ignored. In the current study, we employ a microfluidic organ-on-chip platform to elucidate the time and shear stress dependent effects of elevated hydrostatic pressure on endothelial junctional perturbations. We report that short term (1 h) exposure to elevated hydrostatic pressure at high shear stress (0.1 Pa) but not low shear stress (0.01 Pa) caused VE-cadherin to form finger like projections at the cell–cell junctions, and this effect was abrogated upon pharmacologically inhibiting cationic mechanosensitive channels using GsMTx4 peptide. Interestingly, prolonged exposure (24 h) to elevated hydrostatic pressure at low (0.01 Pa) but not high shear stress (0.1 Pa) caused disruption of VE-cadherin at cell–cell contacts and increased its cytoplasmic concentration. Furthermore, we report that this disruption of VE-cadherin was reversible upon pharmacologically inhibiting cationic mechanosensitive channels in a time-dependent manner; wherein after 12 h, we observed VE-cadherin reassemble at the cell–cell junctions. Overall, we demonstrate that cationic mechanosensitive channels play a crucial role in the mechanotransduction of elevated hydrostatic pressure by regulating the VE-cadherin dynamics at cell–cell junctions.

## I. INTRODUCTION

Cells dynamically respond to biochemical factors and a diverse range of mechanical forces that influence critical cellular processes, such as differentiation, proliferation, stiffness, and migration.^1,2^ The vasculature, in particular, experiences three distinct mechanical forces—shear stress, circumferential stretch, and intraluminal hydrostatic pressure. The role of shear stress in facilitating endothelial dysfunction has been well established.^3^ In comparison, despite having a significant influence of cell shape, migration, and morphogenesis^4,5^ very sparse information is available on the mechanobiological role of hydrostatic pressure in vascular disease progression.

Abnormal elevations in tissue pressure in several conditions, such as glaucoma,^6^ pulmonary edema,^7^ and acute compartment syndrome,^8^ have been shown to have a significant impact on the microvasculature haemodynamics resulting in endothelial dysfunction^9–11^ and disease progression. However, the mechanisms by which endothelial cells sense and respond to increases in hydrostatic pressure are largely unknown. Investigations into the distinct effects of hydrostatic pressure on endothelial cells are required to shed light on several physiological and pathological vascular disease mechanisms.

Junctional contacts in endothelial cells are predominantly mediated by vascular endothelial cadherins (VE-cadherin) in conjunction with other adhesion proteins, such as actin cytoskeleton, occludins, claudins, etc.^12^ VE-cadherin (also known as CD144) is a transmembrane adherens junction protein that has been extensively studied for its role in regulating vascular junctional integrity.^13,14^ Actin filaments, by interacting directly with VE-cadherin cytoplasmic tail via β- and α-catenins, provide structural framework and maintain endothelial barrier function.^15^ In response to mechanical stimulus, the actomyosin cytoskeletal complex can generate transmembrane forces, which regulates the distribution of VE-cadherin at the cell–cell junctions and, thus, influencing the strength and stability of the cell–cell junctions.^16^

Organ-on-chip systems have emerged as powerful tools for recapitulating key features of vascular biology *in vitro* with unprecedented spatial and temporal resolution.^17^ By enabling precise control over the cellular microenvironment, such as shear stress, hydrostatic pressure, and biochemical gradients, microfluidics offers a physiologically relevant platform to investigate endothelial behavior and vascular pathophysiology in a high-throughput fashion.^18,19^ The design flexibility of microfluidics enables fabrication of complex geometries such that the cells can be exposed to varying shear stresses within the same device. Chau *et al.* employed one such multi-shear device capable of exposing human umbilical vein endothelial cells (HUVECs) to ten different shear stress levels over two orders of magnitude (0.07 and 13 Pa).^20^ Past studies using microfluidics have shown that changes in shear stress can significantly alter VE-cadherin junctional distribution and hence impact endothelial barrier function which in turn is influenced by oxidative stress and inflammation.^21,22^ For instance, Miao *et al.* observed continuous VE-cadherin/β-catenin distribution at cell–cell junctions in response to laminar shear stress, whereas oscillatory shear stress demonstrated discontinuous VE-cadherin junctions.^23^ Similarly past microfluidic studies have demonstrated the impact of shear stress (duration,^24^ pulsatility,^25^ and disturbances^26^) on actin cytoskeletal remodeling.^27,28^ In comparison, very few studies^29–31^ have reported on the influence of isotropically applied hydrostatic pressure on VE-cadherin junctional dynamics, especially in response to the contractile forces generated by the actomyosin cytoskeleton which can lead to VE-cadherin disruption at cell–cell junctions.^32^ In one such study, Ohashi *et al.* reported that exposure to high hydrostatic pressure (6666.12, 13 332.2, and 19 998.4 Pa) for 24 h pressure led a marked decrease in VE-cadherin expression compared to static controls although the mechanism for this observed phenomenon was not further investigated.^30^ In contrast, Muller-Marschhausen *et al.* reported that hydrostatic pressure (147 Pa) application produced a protective effect on endothelial cells by reducing the loss of VE-cadherin in response to EGTA, a calcium chelation agent.^29^ Thus, despite being a critical driver of many conditions, there is no clear consensus on the effects of hydrostatic pressure on endothelial cells. Also, these past studies, although very informative, consisted of static cultures and did not study the combined influence of hydrostatic pressure at different shear stresses.

Physiological shear stresses can vary largely across the vascular tree ranging from large elastic arteries, which experience a wall shear stress in the range of 1–7 Pa to post-capillary venules, which experience a wall shear stress of 0.1–0.5 Pa.^33,34^ Capillary pressure at the arterial end ranges between ∼4266 and 4799 Pa^35^ and at the venous end ranges between ∼1600 and 2666 Pa,^36^ while the mean capillary pressure ranges between ∼2666 and 4000 Pa^37,38^ depending upon the resistance of pre- to postcapillary vessels.^38^ Compared to the number of studies that have focused on the higher “arterial” shear stress levels, few studies have explored the impact of low shear stresses (<0.5 Pa^2,39^) despite having implications in the pathophysiology of many occlusive vascular conditions.^40,41^ Especially in conditions, such as glaucoma, acute compartment syndromes which involve not just impaired perfusion^42,43^ (and hence low shear stresses) but also elevated hydrostatic pressure in the blood vessels (resulting from microvascular occlusion^10,44^), the resulting microvascular dysfunction cannot be attributed to low shear stress alone but also to the influence of elevated hydrostatic pressure. In this respect, investigating the impact of elevated hydrostatic pressure at low shear stresses would shed light on previously unknown endothelial mechanotransduction mechanisms. Toward this goal, this study employed an easy-to-implement microfluidic blood vessel model to investigate the short-term and long-term combined influence of shear stress and elevated hydrostatic pressure on VE-cadherin and actin dynamics.

## II. RESULTS

### A. Elevated hydrostatic pressure at high shear stress produces punctuate VE-cadherin junctions

Primary human umbilical vein endothelial cells (HUVECs) were seeded at a density of 10^5^ cells/device (0.2% gelatin coated) and incubated at 37 °C and 5% CO_2_ for 2 h after which perfusion was started. Syringe pumps were used to expose the cells for 1 h to two flow rates, namely, 1.3 *μ*L/min for low shear stress (0.014 Pa) and 13 *μ*L/min for high shear stress (0.14 Pa) experiments after which cells were fixed and immunostaining was performed for VE-cadherin, actin, and nuclei. To apply a hydrostatic pressure (HP) head to both shear stress conditions, the height of the outlet tube was raised to 40 cm to that of the inlet [refer to the Methods section, Figure 5(B)]. This increased the inlet pressure from 4.71 Pa (low shear stress without HP) to 3928.7 Pa (low shear stress with HP) in the low shear stress conditions and from 47.1 Pa (high shear stress without HP) to 3971.1 Pa (high shear stress with HP) in the high shear stress conditions, respectively (refer to Table I in the Methods section). For the rest of the article, the following conventions for each flow condition would be used: low shear stress with HP (SS low + HP), high shear stress with HP (SS high + HP), low shear stress without HP (SS low only), and high shear stress without HP (SS high only). Control cells consisted of cells cultured within the microfluidic device under static conditions with no flow for 1 h.

**TABLE I.** Flow rates for different test conditions and their associated shear stress and pressure head (at inlet) values.

	Flow rate (*μ*L/min)	Wall shear stress (Pa)	Pressure (Pa) at inlet (∼)
SS high only	13	0.14	47.1
SS low only	1.3	0.014	4.71
SS high+HP	13	0.14	3971.1
SS low+HP	1.3	0.014	3928.7

**FIG. 1.**
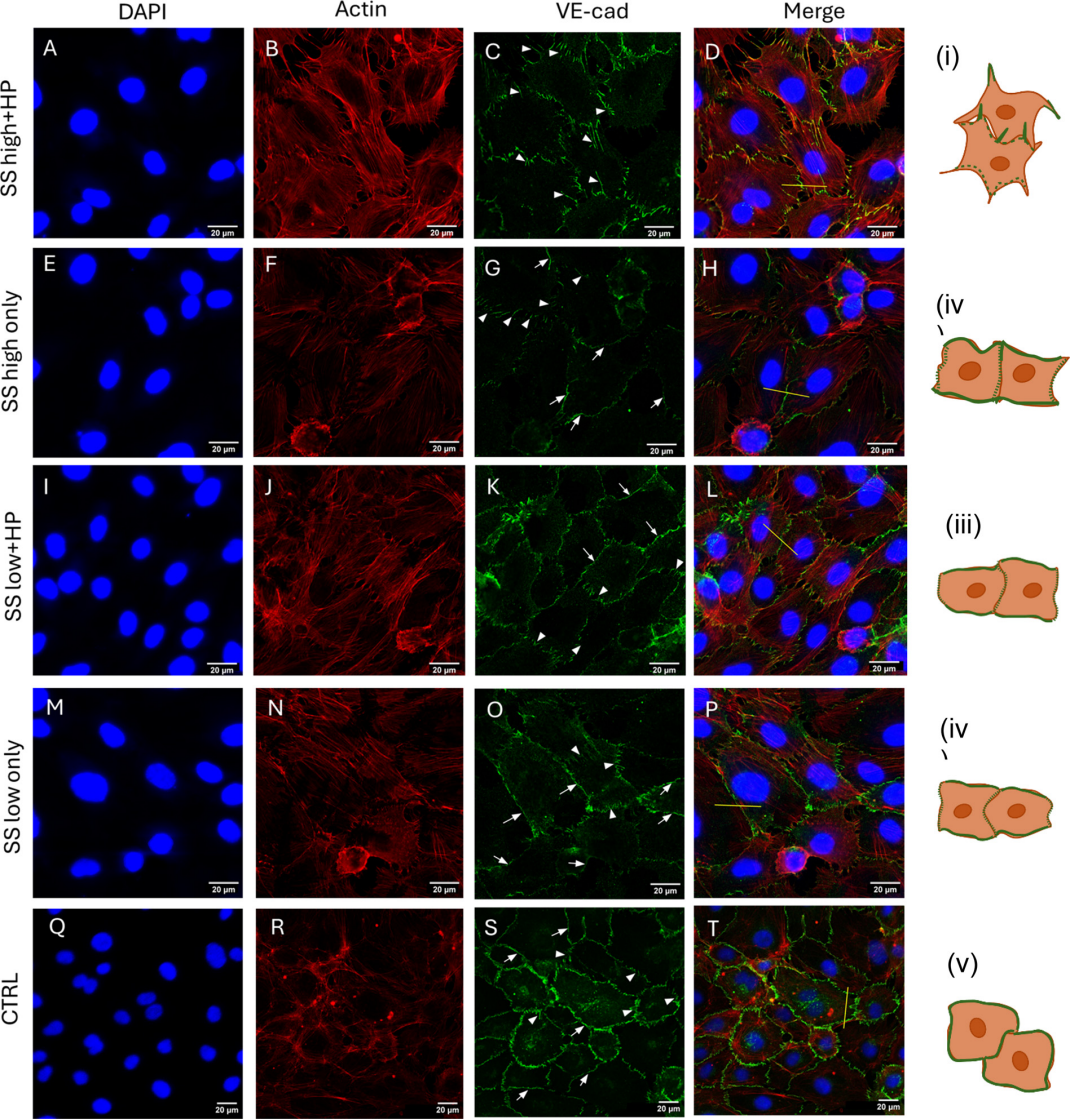

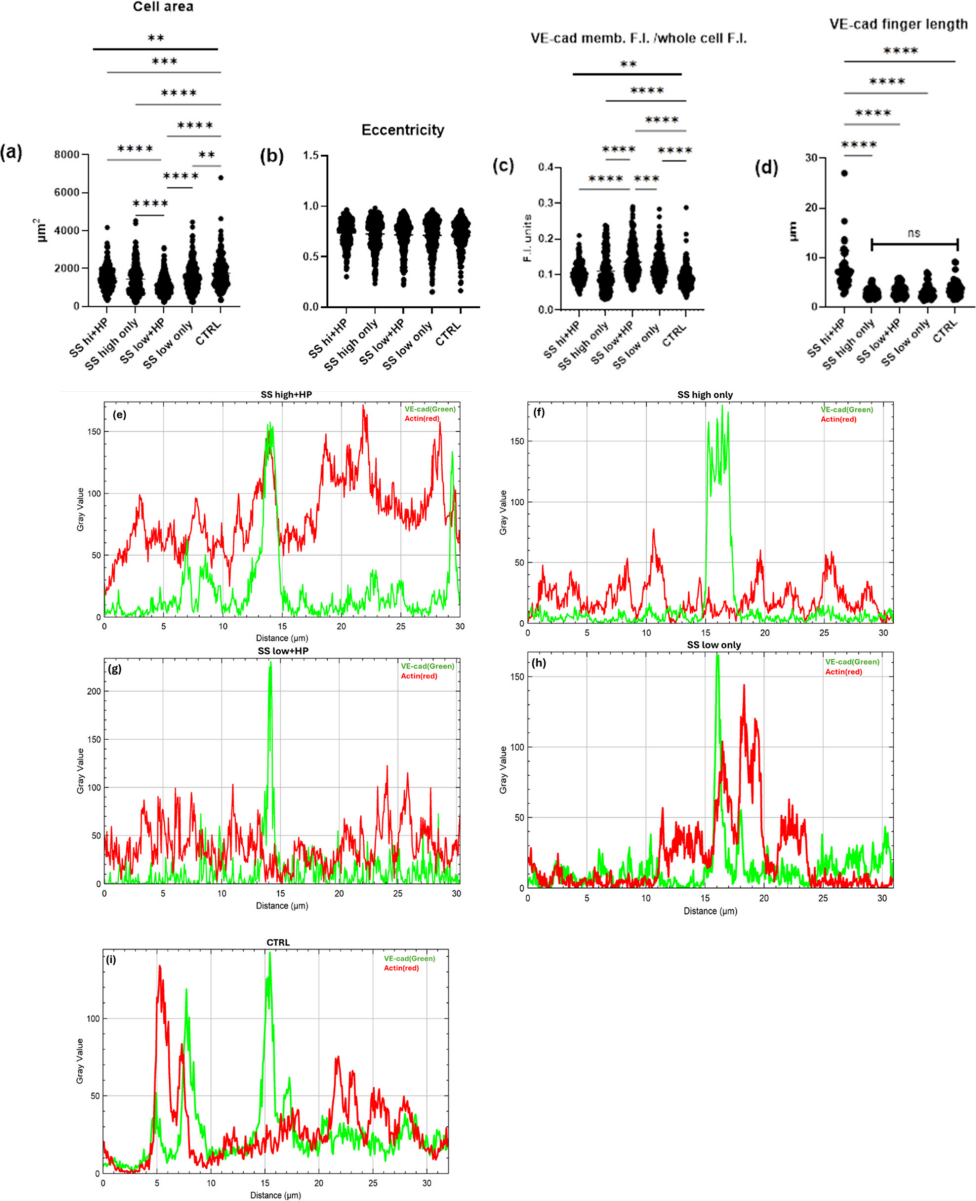
Elevated hydrostatic pressure exposure for 1 h at high shear stress causes VE-cadherin finger formation at cell–cell junctions. (A)–(D) Cells exposed to SS high + HP exhibited VE-cadherin fingers at the cell–cell junctions (denoted by white arrow heads) that highly co-localised with actin protrusions engulfing into neighboring cells. (E)–(H) In contrast, cells exposed to SS high only demonstrated predominantly continuous VE-cadherin pattern (denoted by white arrows) at cell–cell junctions with some junctions demonstrating smaller VE-cadherin fingers (compared to the SS high + HP VE-cadherin fingers). In these junctions, VE-cadherin demonstrated minimal co-localization with actin fibers of the same z-plane. (I)–(L) Similar to SS high only conditions, cells exposed to SS low + HP conditions demonstrated continuous VE-cadherin patterning at cell–cell junctions, especially on the sides, whereas the front and back of cells expressed relatively smaller VE-cadherin fingers. (M)–(P) Cells exposed to SS low only demonstrated continuous VE-cadherin cell–cell junctions with smaller VE-cadherin fingers (similar to SS high only, SS low + HP) at some junctions. In these conditions, VE-cadherin demonstrated higher co-localization with actin compared to SS low + HP and SS high only. (Q)–(T) Control cells demonstrated typical endothelial static cell phenotype with continuous VE-cadherin pattern at cell–cell junctions and thick actin stress fibers across the cell body. (a) Average cell area, (b) cell eccentricity, (c) ratio of membrane VE-cadherin F.I/whole cell VE-cadherin F.I. (cytoplasm + membrane F.I.), (d) average VE-cadherin finger length (e)–(i) line plots of VE-cadherin (green) and actin (red) for SS high + HP, SS high only, SS low + HP, SS low only, and CTRL, respectively, based on the yellow line (drawn across cell–cell junctions) drawn in D, H, L, P, and T. (i)–(v) Schematic representation of cell morphologies observed in different conditions. Green borders at the cell membrane in the schematic represents VE-cadherin. N = 3, at least 50 cells were analyzed per repeat. Flow direction: top to bottom. White arrows: continuous VE-cadherin junctions and white arrow heads: VE-cadherin fingers. Mag = 63× (A)–(L) and 40× (M)–(P).

All conditions demonstrated significantly smaller cell area compared to the control conditions with SS low + HP demonstrating the smallest cell area, as shown in Fig. 1(a). There was no significant difference between SS high + HP and SS high only implying that at 1 h exposure, addition of HP did not influence the cell area. In contrast, in the low shear stress conditions, there was a significant difference in the cell area between SS low + HP and SS low only. These results demonstrate that the effect of HP on cell area is shear stress dependent. Cells in all four flow conditions demonstrated a polygonal morphology with no difference in eccentricity compared to the control, as shown in Fig. 1(b), although the cells exposed to SS high + HP conditions demonstrated a more irregular shape compared to the other conditions as shown in Figs. 1(A)–1(D). All four flow conditions exhibited thick stress fiber formation that traversed the entire cell body. There was no particular orientation of actin fibers in the direction of flow with different areas of the channels demonstrating different orientation angles (refer the supplementary Fig. S1 for orientation angles at different locations of the channel). This lack of alignment is possibly due to the short exposure time of 1 h as endothelial cells generally take between 6 and 24 h to exhibit alignment and elongation in the direction of the flow.^45,46^

VE-cadherin presence at cell–cell junctions was quantified by measuring the fluorescence intensity (F.I.) of VE-cadherin staining at cell–cell junctions in individual z-planes. We used the following formulas to calculate VE-cadherin at cell–cell junctions: (membrane VE-cadherin F.I.)/(whole cell VE-cadherin F.I.). While there was no significant difference in the junctional VE-cadherin F.I. between SS high + HP and SS high only, in the low shear stress conditions, SS low + HP demonstrated significantly higher junctional VE-cadherin F.I. compared to SS low only and control, as shown in Fig. 1(c). All conditions demonstrated higher VE-cadherin F.I. compared to the control conditions.

We then investigated VE-cadherin phenotypic expression pattern and its co-localization with actin at the cell–cell junctions in different conditions. As shown in Figs. 1(A)–1(D), cells in the SS high + HP conditions demonstrated a punctuate VE-cadherin pattern, wherein fingerlike VE-cadherin structures protruded into the neighboring cells as shown in Fig. 1(C) using the white arrowheads. VE-cadherin and actin co-localization were then analyzed by drawing line plots across cell-cell junctions [yellow lines in Figs. 1(D), 1(H), 1(L), 1(P), and 1(T)]. As shown in Fig. 1(e) line plot, there was extensive spatial co-localization of VE-cadherin and thick actin stress fibers in these punctuate VE-cadherin junctions compared to the other flow conditions and the control. Cells in SS high only, SS low + HP, SS low only, and control conditions demonstrated relatively lesser co-localization of VE-cadherin with actin fibers of the same z-plane compared to SS high + HP as demonstrated by the line plots in Figs. 1(f)–1(i), respectively. Such VE-cadherin/actin rich protrusions at cell–cell junctions have been referred to by several studies under different names, such as zipper-like adherens junctions,^47^ punctuate adherens junctions,^48^ and serrated adherens junctions. For the rest of this article, we will to refer to these VE-cadherin-actin rich protrusions as VE-cadherin fingers, a term previously employed by Hayer *et al.*^49^

In contrast to the prominent VE-cadherin fingers observed in the SS high + HP conditions, cells in all other flow conditions, namely, SS high only, SS low + HP, SS low only, as well as control cells demonstrated continuous VE-cadherin patterns, mostly at the sides of the cells as shown in Figs. 1(G), 1(K), 1(O), and 1(S), respectively (denoted by white arrows). A few cell-cell junctions in the other three flow conditions exhibited small, immature VE-cadherin fingers especially at the top and bottom of the cells as shown by the white arrow heads in Figs. 1(G), 1(K), and 1(O) [these small VE-cadherin fingers were observed in control conditions too as seen in Fig. 1(S)]. As shown in Fig. 1(d), the VE-cadherin fingers in SS high + HP were significantly longer compared to SS high only condition (refer to supplementary material Fig. S2 for a direct comparison of VE-cadherin fingers between the SS high + HP and SS high only) as well as the low shear stress and control conditions, thus implying that the addition of HP to SS high condition has a significant impact on VE-cadherin finger formation at the cell–cell junctions. These results indicate that the mechanotransduction from a 1 h elevated hydrostatic pressure exposure is moderate at low shear stresses (indicated by the presence of continuous VE-cadherin at junctions) but can have a significant effect at higher shear stresses (indicated by the presence of distinct VE-cadherin fingers at junctions).

### B. Inhibiting mechanosensitive channels leads to increased VE-cadherin localization at cell–cell junction

Since cation permeable mechanosensitive channels, such as TRPC1, TRPC6, and piezo-1, are known to be extremely sensitive to any mechanical force experienced by the endothelial membrane lipid bilayer,^50–52^ we next investigated the impact of pharmacologically blocking these mechanosensitive channels using a small peptide inhibitor GsMTx4 on the VE-cadherin junctional dynamics at elevated pressure. The culture conditions and the flow rates used in this set of experiments were identical to that described in the 1 h experiments. All four flow conditions SS high + HP, SS high only, SS low + HP, and SS low only were treated for 1 h to media containing 5 *μ*M GsMTx4, which is known to selectively inhibit cation permeable mechanosensitive channels at micromolar concentrations.^53^ Control cells consisted of cells cultured within the microfluidic device under static conditions with no flow and no GsMTx4 for 1h.

**FIG. 2.**
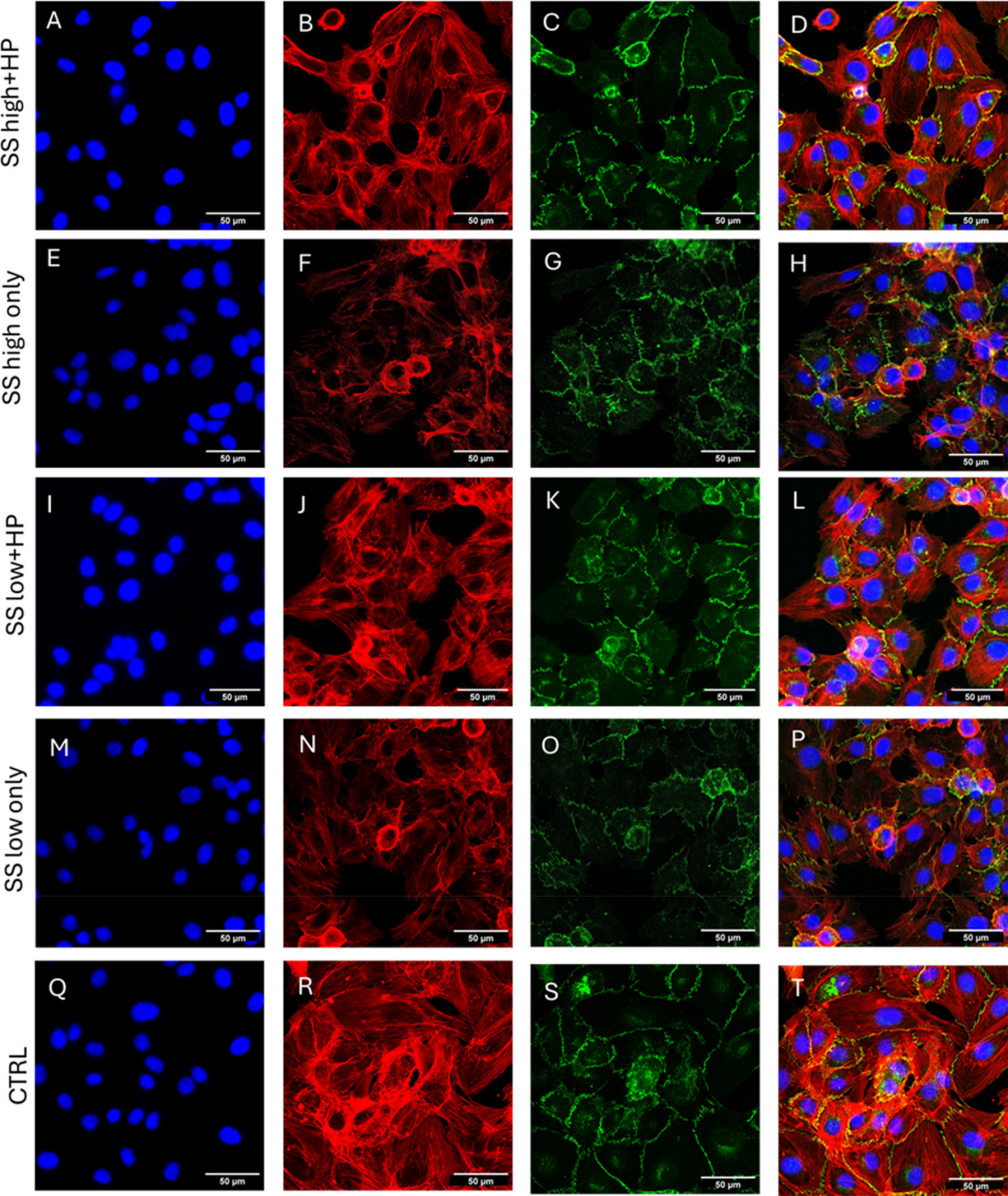

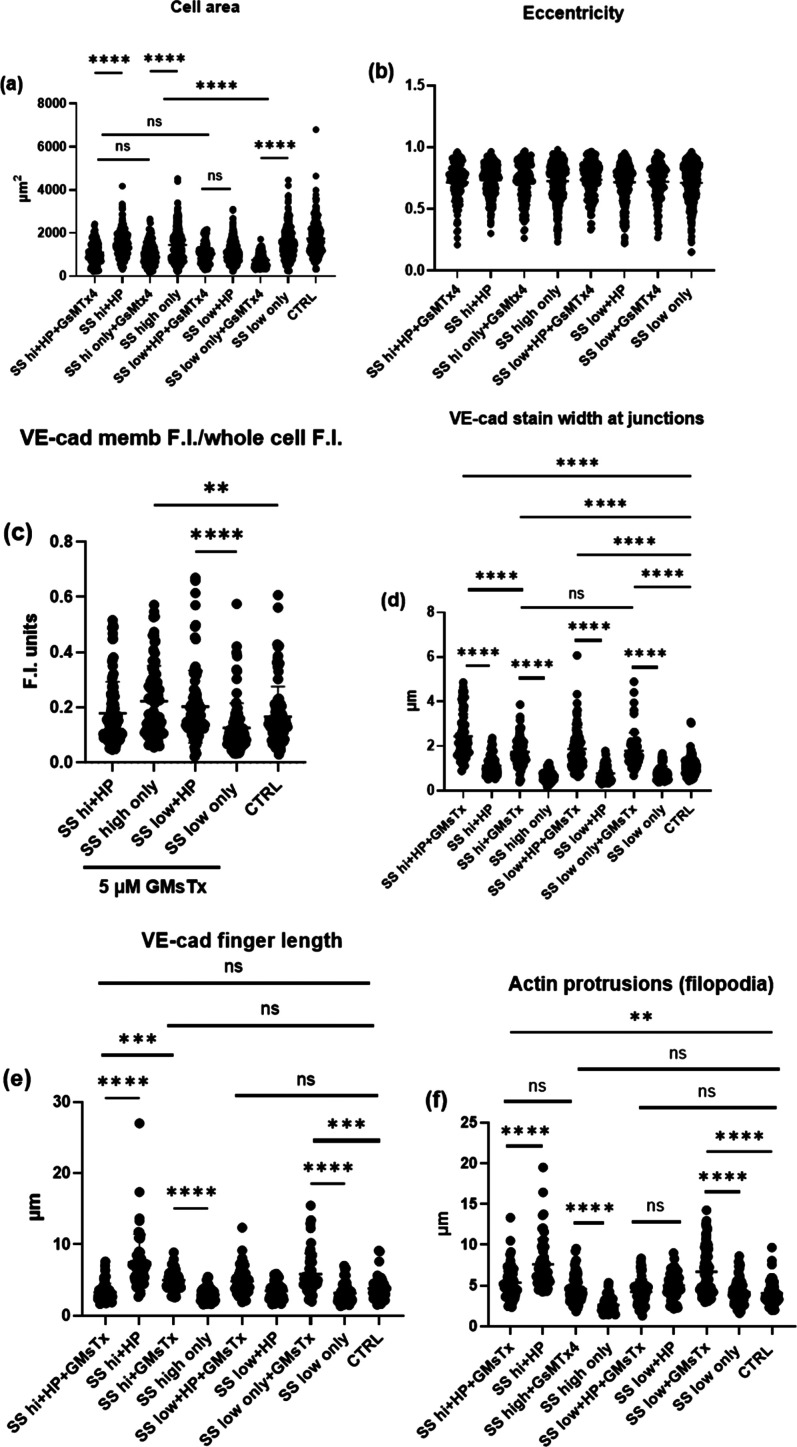
Influence of inhibiting mechanosensitive channels on the effect of short term elevated hydrostatic pressure exposure. (A)–(D) SS high + HP in the presence of GsMTx4 demonstrated thick, continuous VE-cadherin junctions as well as VE-cadherin fingers at the cell membrane although the number and length of VE-cadherin fingers decreased compared to the SS high + HP conditions. (E)–(H) SS high only conditions demonstrated a decrease in cell area in the presence of GsMTx4 but an increase in VE-cadherin finger, filopodial protrusion length as well as VE-cadherin stain width at the cell–cell junctions. (I)–(L) SS low + HP in the presence of GsMTx4 demonstrated a similar pattern of thick VE-cadherin stain width at the cell–cell junctions compared to the non-inhibited conditions. (M)–(P) SS low only condition in the presence of GsMTx4 also demonstrated increased VE-cadherin finger length, filopodial protrusion length, and increased VE-cadherin stain width at junctions compared to the non-inhibited conditions. (Q)–(T) Control cells demonstrated typical endothelial static cell phenotype with continuous VE-cadherin pattern at cell–cell junctions (a) average cell area, (b) cell eccentricity, (c) ratio of membrane VE-cadherin F.I/whole cell VE-cadherin F.I. (cytoplasm + membrane F.I.), (d) average VE-cadherin stain width at cell–cell junctions, (e) average VE-cadherin finger length, and (f) average filopodial protrusion length. N = 3, at least 50 cells were analyzed per repeat. Flow direction: Top to bottom. Mag = 40×.

Blocking mechanosensitive channels via GsMTx4 treatment led to a significant reduction in the average cell area in all four flow conditions compared to the non-inhibited conditions except in SS low + HP as shown in Fig. 2(a) (all inhibited conditions were significant compared to the control as well). There was no change in eccentricity between the different conditions as shown in Fig. 2(b) and cells in all the four flow conditions exhibited thick, actin stress fibers. When analyzed for VE-cadherin F.I. at cell-cell junctions (VE-cadherin membrane F.I/whole cell F.I), similar to the 1 h normal treatment, SS high + HP and SS high only did not demonstrate any significant difference whereas SS low+HP demonstrated significantly higher junctional VE-cadherin compared to SS low only as shown in Fig. 2(c). With respect to control, only SS high conditions demonstrated a significant difference.

Interestingly, there was a significant increase in the VE-cadherin recruitment at the cell–cell junctions in all flow conditions as evidenced by the increase in the width of VE-cadherin staining at the cell–cell junctions compared to the non-inhibited conditions, as shown in Fig. 2(d). In addition, while there was a significant decrease in the VE-cadherin finger length in SS high+HP, other conditions like SS high only and SS low only demonstrated an opposite effect exhibiting an increase in VE-cadherin finger length, as shown in Fig. 2(e). Moreover, SS low+HP did not demonstrate any significant difference in VE-cadherin finger length compared to the non-inhibited conditions. A very similar trend was observed for filopodia protrusion length, as shown in Fig. 2(f), wherein SS high+HP condition demonstrated a significant decrease, SS high only and SS low only demonstrated a significant increase and SS low+HP did not exhibit any difference in filopodial length when compared to the non-inhibited conditions. These observations were interesting as it demonstrated that inhibiting mechanosensitive channels during the application of a hydrostatic pressure can alter VE-cadherin finger and filopodial protrusion lengths differently: it can either lead to decreased VE-cadherin finger/filopodial length as observed in SS high+HP or it does not alter the VE-cadherin finger/filopodial length as observed in SS low+HP, whereas the removal of hydrostatic pressure in SS high and SS low only conditions caused an increase in the VE-cadherin finger/filopodial length. This suggests that the cationic mechanosensitive channels play a key role in the mechanotransduction of hydrostatic pressure to regulate VE-cadherin/actin protrusions at cell–cell junctions.

### C. Prolonged exposure to elevated hydrostatic pressure disrupts junctional VE-cadherin but only at low shear stress

We next investigated the long-term effects of elevated hydrostatic pressure application at both shear stresses. The culture conditions and the flow rates for both shear stresses were identical to that of the 1 h exposure conditions except that the exposure time was 24 h post which the cells were fixed, and immunostaining was performed for VE-cadherin, actin, and nuclei. Since there was no difference between SS high+HP and SS high only, in terms of cell area, eccentricity, and junctional VE-cadherin F.I. and since SS low+HP and SS low only demonstrated significant differences in cell area and VE-cadherin F.I. [refer Figs. 1(a)–1(c)], we decided to focus on three flow conditions for the 24 h experiments, namely, SS high+HP, SS low+HP, and SS low only. Control cells consisted of cells cultured within the microfluidic device under static conditions with no flow for 24 h.

**FIG. 3.**
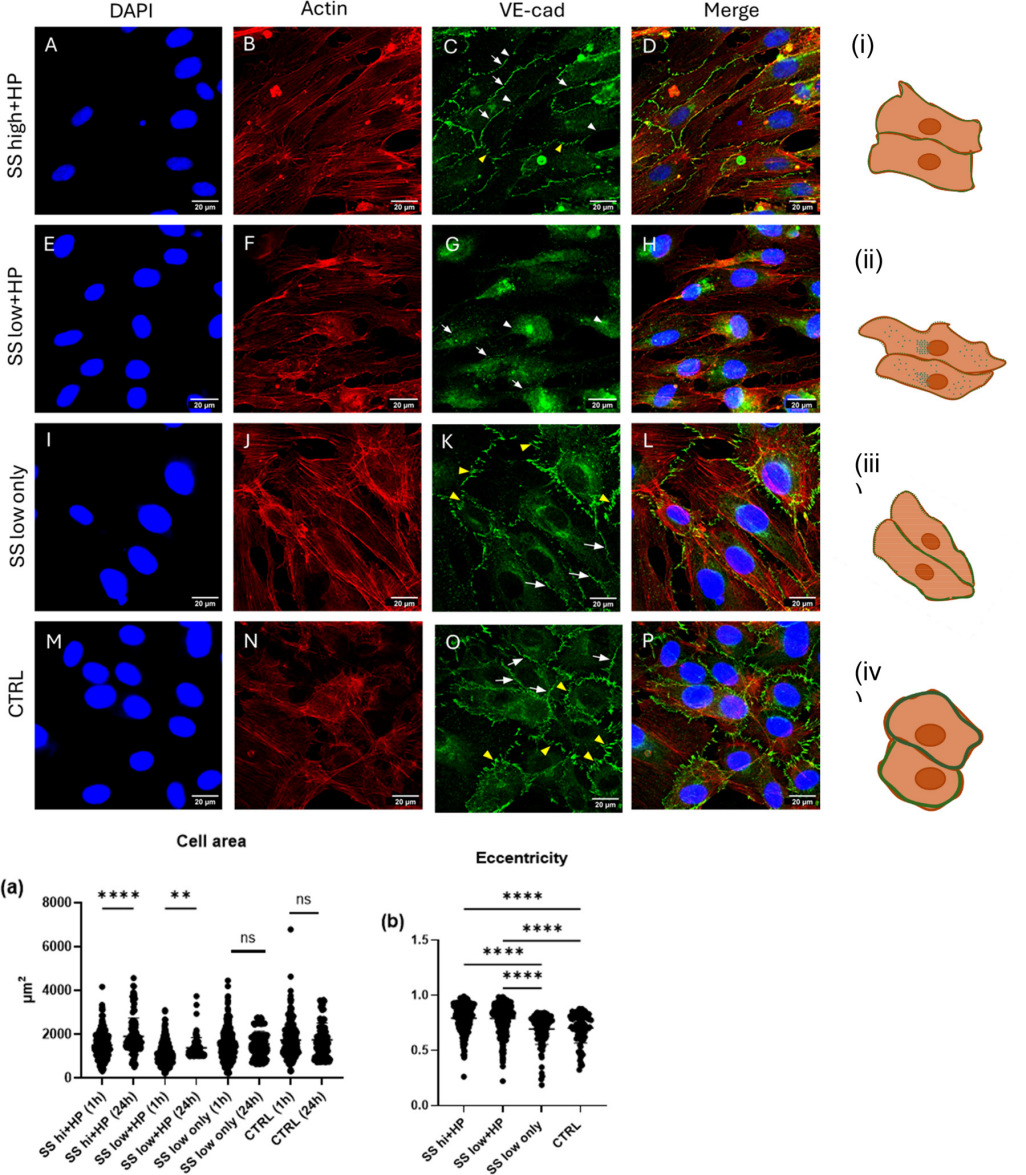

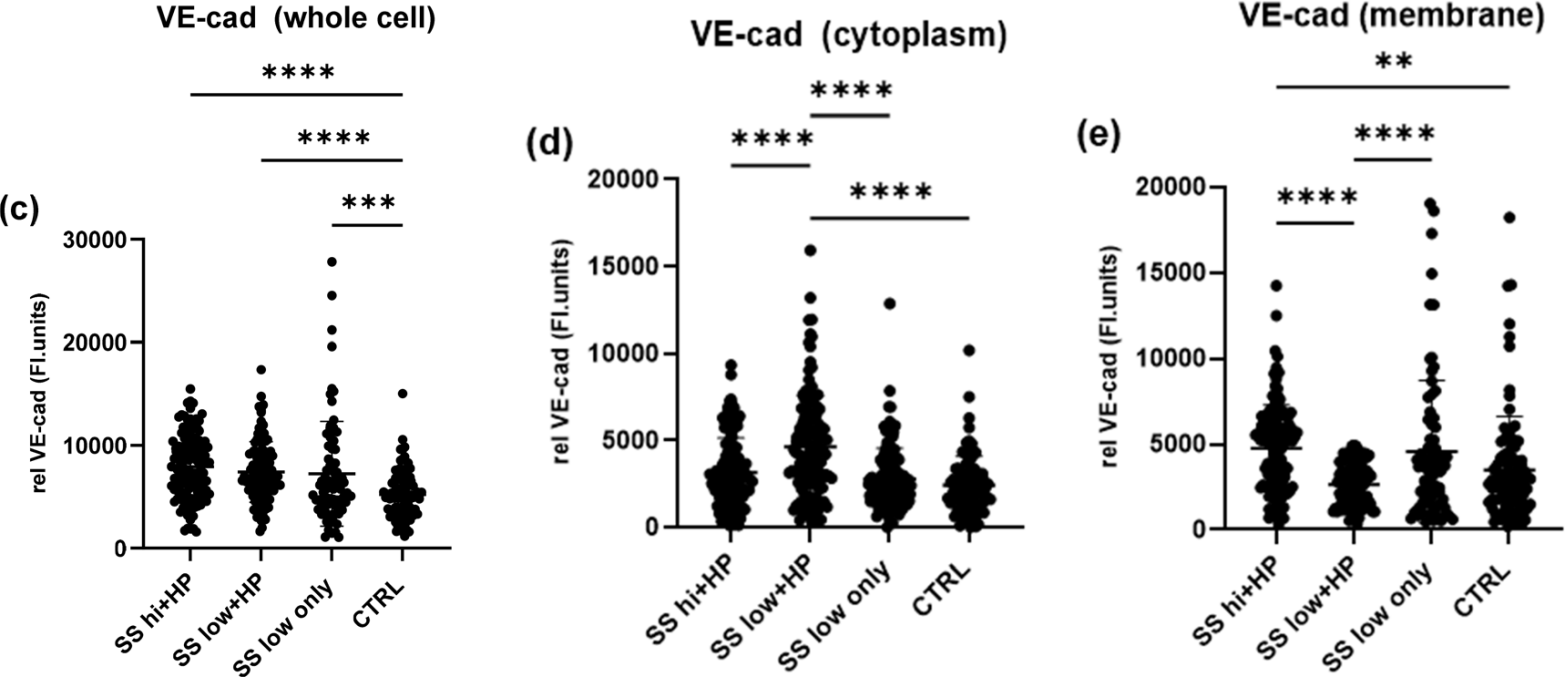
Long term elevated hydrostatic pressure exposure for 24 h at low shear stress causes VE-cadherin disruption at cell–cell junctions. (A)–(D) Cells exposed to SS high+HP demonstrated mostly continuous junctions (white arrows) with intermittent disruptions (white arrow heads) of VE-cadherin. A few cells also exhibited small VE-cadherin fingers (yellow arrowhead) (E)–(H) Cells exposed to SS low+HP demonstrated an almost complete disruption of VE-cadherin at the cell-cell junctions and an increased presence in the cytoplasmic area (white arrow heads). A few junctions demonstrated a very thin lining of VE-cadherin (white arrows). Although there was no difference in relative VE-cadherin F.I. between the flow conditions when analyzing whole cells, there was a marked difference in the cytoplasmic and membrane F.I., with SS low+HP exhibiting higher cytoplasmic VE-cadherin staining compared to the membrane, thus indicating a loss of junctional VE-cadherin. (I)–(L) Cells exposed to SS only demonstrated mostly continuous junctions (white arrows) with some junctions exhibiting VE-cadherin fingers (yellow arrow heads) (M)–(P) static control demonstrated continuous junctional staining (white arrows) with VE-cadherin fingers (yellow arrow heads) in some junctions similar to that of the SS low only cells. (a) Average cell area comparison between the 1 and 24 h exposure times, (b) cell eccentricity comparison between the 1 and 24 h exposure times, (c) relative VE-cadherin F.I. in whole cell, (d) relative VE-cadherin F.I. in the cell cytoplasm, and (e) relative VE-cadherin F.I. in the cell membrane. (i)–(iv) Schematic representation of cell morphologies observed in different conditions. Green borders at the cell membrane in the schematic represent VE-cadherin. N = 3, at least 50 cells were analyzed per repeat. Flow direction: Left to right. Mag = 63×.

Cells exposed to SS high+HP for 24 h demonstrated an elongated morphology, as shown in Figs. 3(A)–3(D), with an increased average cell area compared to the 1 h exposure conditions as shown in Fig. 3(a). Furthermore, SS high+HP demonstrated significantly less circularity compared to the control cells as well as the SS low only conditions as quantified in Fig. 3(b). Cells also demonstrated aligned actin stress fibers covering the entire cell body and forming extensive connections with the neighboring cells with increased actin/VE-cadherin co-localization at the cell–cell contacts (line plots quantification in supplementary material Fig. S3). VE-cadherin clustered predominantly at the cell membrane as continuous junctions [Fig. 3(C), white arrows] with some intermittent disruptions [Fig. 3(C), white arrowheads] and some cells exhibiting immature VE-cadherin fingers [Fig. 3(C), yellow arrowheads]. SS low only and control cells did not demonstrate any changes in the cell area or eccentricity, as shown in Figs. 3(a) and 3(b), respectively. Both conditions demonstrated continuous VE-cadherin patterns [white arrows in Figs. 3(K) and 3(O)] with a few junctions exhibiting VE-cadherin fingers [yellow arrow heads in Figs. 3(K) and 3(O)].

In comparison to SS high+HP conditions, cells exposed to SS low+HP for 24 h demonstrated a substantial disruption of VE-cadherin at the cell–cell junctions with only a faint, thin VE-cadherin lining at cell–cell contacts [Fig. 3(G), white arrows]. SS high+HP and SS low+HP did not demonstrate any significant difference in whole cell VE-cadherin F.I., as shown in Fig. 3(c). Interestingly, in the SS low+HP conditions, there was an increased localization of VE-cadherin in the cell cytoplasm [Fig. 3(G), white arrowheads] and a lack of VE-cadherin at the cell–cell junctions. Figures 3(d) and 3(e) quantitatively demonstrate this increased presence of cytoplasmic VE-cadherin and its loss at cell membrane, respectively. This suggests that prolonged exposure to elevated hydrostatic pressure under low shear stress can lead to a significant disassembly of VE-cadherin at cell–cell junctions.

### D. Loss of junctional VE-cadherin is reversible upon inhibiting mechanosensitive channels

We next investigated if the loss of VE-cadherin at cell–cell junctions observed in the 24 h exposure to SS low+HP condition could be reversed by blocking the mechanosensitive channels. For these experiments, the cells were first exposed to SS low+HP for 24 h after which they were then post-treated to a growth medium containing 5 *μ*M GsMTx4 for either 4 or 12 h. After this, the cells were fixed, and immunostaining was performed for VE-cadherin, actin, and nuclei.

**FIG. 4.**
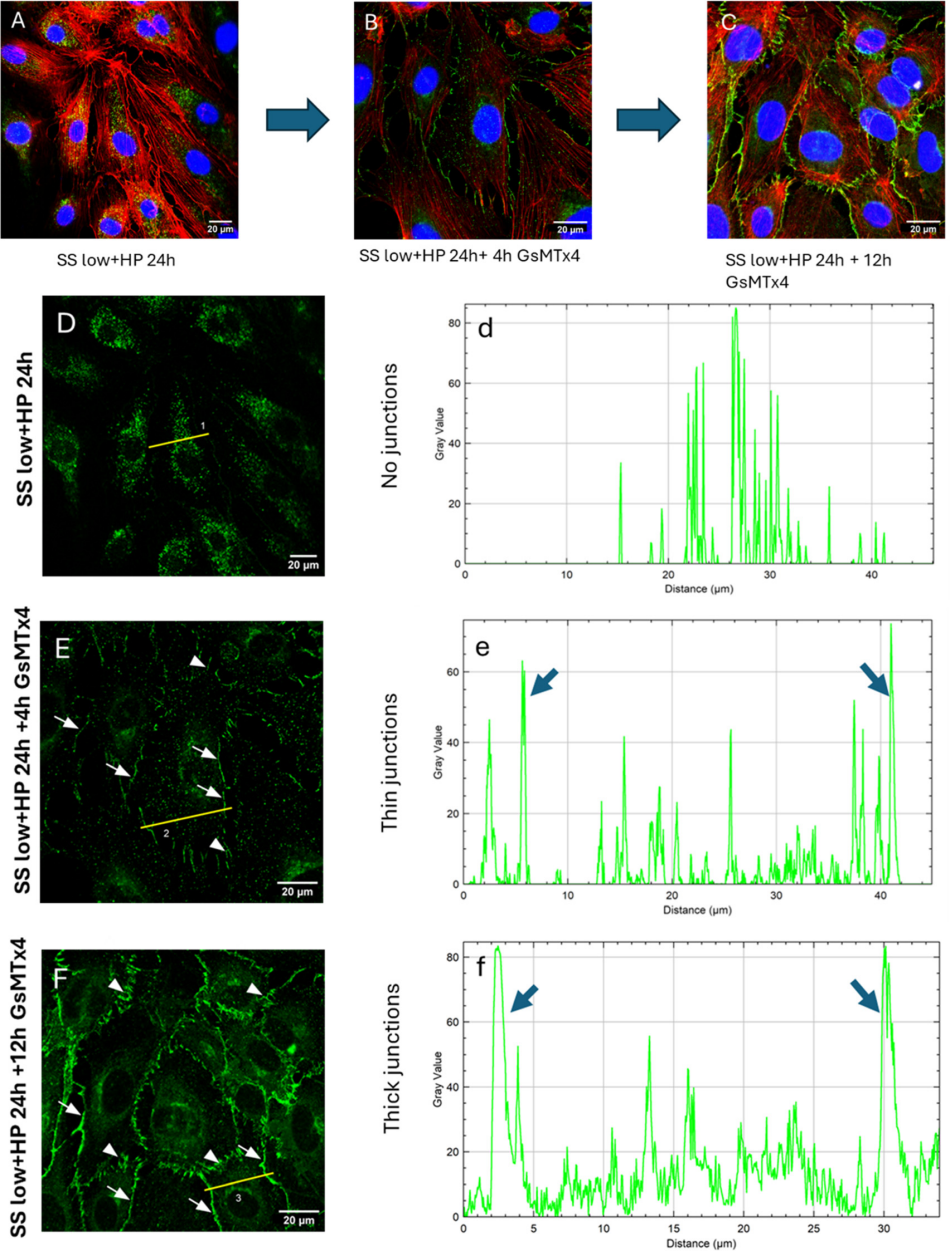
Disruption of VE-cadherin at cell–cell junctions is reversible and dependent on mechanosensitive channels. (A) Cells exposed to SS low+HP for 24 h, (B) Cells exposed to SS low+HP for 24 h followed by treatment to 5 *μ*M GsMTx4 for 4 h, and (C) Cells exposed to SS low+HP for 24 h followed by treatment to 5 *μ*M GsMTx4 for 12 h. It can be observed that cells treated to GsMTx4 for 4 h post SS low+HP exposure demonstrated reduced VE-cadherin cytoplasmic presence and exhibited development of thin, continuous VE-cadherin junctions [denoted by the white arrows in (E) and blue arrows in (e)]. Upon treating the cells to 12 h of GsMTx4 post SS low+HP exposure, the cells demonstrated the formation of thick, continuous junctions [denoted by the white arrows in (F) and blue arrows in (f)] as well as immature, VE-cadherin fingers [denoted by the white arrowheads in (F)]. Flow direction: Top to bottom. Mag = 40× (A) and (D) and 63× (B), (C), (E), and (F).

As it can be noticed from Figs. 4(A)–4(C), treating cells to 5 *μ*M GsMTx4 for 4 h led to partial reassembling of VE-cadherin at cell–cell junctions, whereas a 12 h treatment to 5 *μ*M GsMTx4 led to formation of prominent continuous VE-cadherin junctions along the cell–cell contacts. Furthermore, the VE-cadherin line plots in Figs. 4(d)–4(f) demonstrated the gradual formation of continuous VE-cadherin junctions with increasing GsMTx4 post-treatment time. Figure 4(E) and the corresponding line plot Fig. 4(e) demonstrated the formation of thin, interrupted VE-cadherin junctions [white arrows in Fig. 4(E)] and decreased presence of cytoplasmic VE-cadherin after 4 h GsMTx4 post-treatment compared to Fig. 4(D) and line plot Fig. 4(d) which exhibited a loss of junctional VE-cadherin and an increased cytoplasmic VE-cadherin presence. Figure 4(F) and line plot Fig. 4(f) demonstrated that after 12 h GsMTx4 post-treatment, thick, continuous VE-cadherin junctions [white arrows in Fig. 4(F)] with no significant cytoplasmic VE-cadherin presence was observed. Furthermore, immature, short VE-cadherin fingers were also observed after the 12 h GsMTx4 post-treatment [white arrowheads in Fig. 4(F)]. These results demonstrate the crucial role of mechanosensitive channels in regulating VE-cadherin recruitment dynamics at the cell–cell junctions.

## III. DISCUSSION

The pathophysiology of diseases, such as glaucoma and acute compartment syndrome, involves increased pressure experienced by the vasculature which causes microvascular dysfunction and tissue degeneration.^54–56^ In comparison to shear stress, the mechanobiological role of elevated hydrostatic pressure in the progression of vascular dysfunction has surprisingly received limited attention.^57,58^ In the current study, we employed a microfluidic model of the blood vessel to investigate the combined influence of low shear stresses and elevated hydrostatic pressure on endothelial cell behavior. The simplicity of our experimental setup enabled us to expose the endothelial monolayer to an elevated hydrostatic pressure environment in the presence of flow by raising the outlet tube to a height of 40 cm relative to the inlet to add a hydrostatic pressure of ∼4000 Pa. The ease of implementation of this microfluidic setup would allow investigators in any lab to experiment with various pressure regimes by simply altering the height of the outlet tube relative to the inlet to get the desired pressure head. Such a simple system is especially required to promote comparability between studies in the less explored field of hydrostatic pressure induced endothelial mechanotransduction, which has reported on contradicting findings due to variability in the experimental setups.^29,59^

Previous studies have shown elevated hydrostatic pressure to promote endothelial angiogenesis,^60^ tubulogenesis,^61^ proliferation,^62^ and even influence survival of vascular smooth muscle cells.^63^ This is the first study to report on the temporally variable and shear-stress dependent effects of hydrostatic pressure on VE-cadherin and actin dynamics at cell–cell junctions. Our study demonstrated that without the addition of any inflammatory stimuli, a 1 h exposure to elevated hydrostatic pressure at high but not low shear stress led to VE-cadherin fingers at the cell–cell junctions. Such prominent and significantly longer VE-cadherin fingers were not observed in the SS high only cell–cell junctions which substantiates the individual effect of HP on VE-cadherin patterning at junctions. Previously, Huveneer *et al.* reported that the presence of such VE-cadherin fingers, co-localizing with radial actin fibers and vinculin at cell–cell junctions is indicative of a remodeling junction, which is in contrast to a mature, stable junction marked by continuous VE-cadherin that aligned with thick actin fibers but did not overlap with it.^64^ Further investigations into the role of hydrostatic pressure-induced VE-cadherin finger formation and its relevance to junctional remodeling/stability could help shed light on important physiological and pathological processes, such as angiogenesis, inflammatory remodeling, and vascular hyperpermeability. Interestingly, our study also showed that despite demonstrating similar cell confluency, short term exposure to SS high only, SS low + HP, and SS low only demonstrated continuous VE-cadherin junctions, whereas SS high + HP resulted in VE-cadherin finger formation at cell–cell junctions. This observation was contrary to past studies that showed that punctuated VE-cadherin junctions to be a marked feature of sub-confluent cell density and continuous VE-cadherin junctions are characteristic of confluent cell density.^65,66^ A possible explanation for this cell density independent variable VE-cadherin patterning could be that the cells produce the same level of endogenous VE-cadherin irrespective of the cell density (as a part of the cell energy conservation mechanism to avoid the constant synthesis of new proteins^65^) as previously stated.^67^ The type and magnitude of mechanical stimuli would then determine the VE-cadherin patterns i.e., continuous junctions vs finger like projections at the junction. It is also important to note that the effect of hydrostatic pressure is temporally variable. While SS low+HP at 1h formed linear, continuous cell-cell junctions, SS low+HP at 24h demonstrated substantial disruption of VE-cadherin at the cell-cell junctions. This demonstrates that the impact of hydrostatic pressure can have a protective, neutral or disruptive effect depending upon not just the shear stress magnitude but also the exposure time which agrees with previous reports.^29,61^ In an interesting study highlighting the variable temporal effect of hydrostatic pressure, Prystopiuk *et al.* reported a two-phase response of endothelial cells to hydrostatic pressure wherein an acute exposure of 1 h to 13 332.2 Pa did not impact VE-cadherin distribution whereas a chronic exposure of 24 h disrupted VE-cadherin at the membrane which is similar to that observed in the current study.^68^

A number of candidates, such as Ca^2+^ channels,^69^ TRPV1,^70^ and aquaporins,^61^ have been proposed as mechanical sensors of pressure changes though the exact mechanism by which transient changes in hydrostatic pressure are sensed by endothelial cells still eludes cell biologists. In order to investigate the role of mechanosensitive channels in hydrostatic pressure mechanotransduction, we used GsMTx4, an inhibitor of cation-permeable stretch activated channels that has been used in the past mechanotransduction studies to inhibit TRPC1,^71^ TRPC6,^72^ and piezo-1.^73,74^ Short term exposure to GsMTx4 lead to increased accumulation of VE-cadherin at cell-cell junctions in all four flow conditions including both the elevated pressure conditions SS high+HP and SS low+HP. This observation agrees with previous studies wherein elevated hydrostatic pressure caused disruption of VE-cadherin at junctions in a piezo-1 dependent manner and caused hyperpermeability.^75,76^ However, an exact opposite effect was reported by Zhong *et al.* wherein activation of piezo-1 channels protected cells from VE-cadherin internalization and promoted junctional integrity in response to mechanical stretching.^77^ Past studies have also demonstrated the impact of TRPC1 on cytoskeletal reorganization and VE-cadherin degradation at the cell-cell junction via Ca^2+^ influx.^78,79^ Tauseef *et al.* reported that Trpc1(-/-) endothelial cells demonstrated a 2.2-fold increase in VE-cadherin expression and also resisted thrombin induced endothelial permeability.^80^ Since the current study used GsMTx4, a broad inhibitor of mechanosensitive channels, it would be interesting to investigate the role of individual mechanosensitive channels, such as TRPC1 and piezo-1 in regulating the VE-cadherin junctional dynamics. Moreover, the above-mentioned studies also suggest that different mechanical stimuli can impact the junctional integrity differently wherein mechanosensitive channels can be barrier disruptive in response to elevated hydrostatic pressure,^75,76^ whereas they can also demonstrate barrier protective properties in response to cyclic mechanical stretch.^77^

In contrast to 1h short term exposure studies, 24 h long term exposure to elevated hydrostatic pressure led to a significant loss of VE-cadherin at the cell–cell contacts in the low shear stress conditions. Two possible mechanisms could explain this phenomenon: increased hydrostatic pressure could have (1) led to an impaired VE-cadherin recruitment to the cell-cell contacts or (2) led to an internalization of VE-cadherin via endocytosis. Past studies have reported on the effect of mechanical forces such as shear stress on VE-cadherin phosphorylation which promotes VE-cadherin internalization.^81,82^ Internalization mechanisms could involve clathrin (but not caveolae)-dependent endocytosis^83^ or β-arrestin mediated endocytosis of VE-cadherin in clathrin coated vesicles.^84^ In terms of the impaired VE-cadherin membrane recruitment hypothesis, past studies have shown that cytosolic phospholipase A2-α (cPLA2α) plays a crucial role is trafficking adherens junction proteins from the golgi apparatus to the cell membrane.^85,86^ Specifically pertaining to endothelial junctional integrity, one past study demonstrated that abrogation of cPLA2α led to impaired trafficking of VE-cadherin and claudin-5 at the cell–cell contacts and an increased accumulation of both the proteins in the golgi area of the cell.^87^ Further studies would be required to ascertain the exact molecular mechanisms of HP mechanotransduction that caused disruption of VE-cadherin at the cell–cell contacts. Moreover, classical dextran permeability assays^88^ would help deciphering if the observed loss of VE-cadherin at the junctions translated to a significant increase in endothelial permeability or not.

Another surprising observation was that in contrast to SS low+HP which demonstrated a substantial loss of junctional VE-cadherin, SS high+HP demonstrated continuous junctions with some intermittent loss of VE-cadherin at cell-cell contacts. One possible explanation might be that compared to SS low+HP, the 10-times higher shear stress experienced by the SS high+HP cells for a prolonged period of time might have helped in the adaptation of the cells to the influence of HP. Previously, Zhong *et al.* demonstrated that higher shear stresses protected VE-cadherin from internalization and improved availability of VE-cadherin at cell-cell junctions compared to the relatively low shear stresses.^89^ Another interesting study also demonstrated that the levels of VE-cadherin phosphorylation (which makes VE-cadherin more susceptible to internalization) decreased upon increasing the shear stress,^82^ thus demonstrating the VE-cadherin protective effects of higher shear stresses. Further investigations into the individual and combined effects of high shear stress and high pressure using time-lapse imaging would help shed light on the dynamic regulation of junctional VE-cadherin during prolonged exposure to HP.

Interestingly, in the current study, we observed that the loss of VE-cadherin at cell-cell junctions was recovered when treated with GsMTx4 post exposure to SS low+HP for 24 h. This observation is in agreement with one past *in vivo* study which showed that increased vascular pressure post stenotic constriction reduced the expression of VE-cadherin and that this reduction in VE-cadherin levels was abrogated upon blocking piezo-1 mechanosensitive channels using GsMTx4, thus demonstrating the inverse relationship between piezo-1 and VE-cadherin recruitment at cell-cell junctions.^75^ Although deciphering the precise molecular mechanisms behind this relationship between VE-cadherin and the individual mechanosensitive channels, such as TRPC1 and piezo-1, was beyond the scope of the current study, it would nevertheless be interesting to investigate the spatiotemporal relationship between VE-cadherin, individual mechanosensitive channels at elevated hydrostatic pressure to investigate how this distinct mechanical force influences critical processes, such as VE-cadherin recruitment, degradation, and recycling at the cell–cell junctions.

## IV. CONCLUSIONS

This study reports on the temporal and shear stress dependent effects of elevated hydrostatic pressure on the dynamics of VE-cadherin and actin at the endothelial cell–cell junctions using an easy-to-implement microfluidic blood vessel platform. We demonstrate that the magnitude of shear stress influences the response of cells to elevated hydrostatic pressure. We show that short-term 1h exposure to elevated hydrostatic pressure at high shear stress (but not low shear stress) led to the formation of VE-cadherin fingers which highly co-localised with the actin protrusions at the cell-cell contacts. Importantly, these VE-cadherin fingers and actin protrusions were abrogated upon pharmacologically blocking mechanosensitive channels using GsMTx4 which was a novel observation. We then demonstrate that unlike 1h exposure conditions, long term 24 h exposure to low shear stress (but not high shear stress) at elevated hydrostatic pressure led to a significant loss of VE-cadherin at cell–cell junctions. Finally, we also demonstrate that this loss of VE-cadherin at the junctions was reversible upon blocking mechanosensitive channels in a time dependent manner. Overall, we conclude that hydrostatic pressure is a distinct and potent mechanical stimuli that can significantly impact the endothelial cell morphology as well as VE-cadherin/actin stability at cell–cell junctions. Further investigations at the interface of elevated hydrostatic pressure, downstream signaling mediated by mechanosensitive channels, and VE-cadherin/actin dynamics at cell–cell junctions could help elucidate previously unknown mechanisms of vascular remodeling and endothelial dysfunction in the context of diseases involving elevated pressure environments.

## V. METHODS

### A. Cell culture

HUVECs were chosen for this study as they faithfully represent the *in vivo* human endothelium behavior compared to the other endothelial cell lines.^90^ Owing to its physiological relevance, HUVECs have been extensively used in both academia as well as biopharmaceutical industries to investigate vascular pathologies.^91^ HUVECs (PromoCell, Heidelberg, Germany) between passages 3–8 were cultured in M199 growth medium supplemented with 10% fetal bovine serum (FBS) (Gibco™, Sigma-Aldrich, UK) and 15 ml endothelial cell growth supplement mix (Cell applications Inc., Merck, UK) per 500 ml media bottle.

### B. Microfluidic experiments

Ibidi *μ*-slide VI 0.1 (Ibidi GmbH, Gräfelfing, Germany) microfluidic channels (1 mm wide, 100 *μ*m height and 17 mm length) were used for the microfluidic experiments. Prior to seeding, the channels were coated with 0.2% gelatin solution (Sigma Aldrich, Merck Ltd, Dorset, UK) and incubated at 37 °C and 5% CO_2_ for 2 h. Channels were washed with fresh growth medium and HUVECs were gradually introduced into the channels at a density of 10^5^ cells/channel (i.e., 10 *μ*l of HUVECs from a cell suspension of 10^7^ cells/ml) without forming air bubbles. Cells were observed under the brightfield microscope (EVOS M3000, Invitrogen, Fisher Scientific, Loughborough, UK) to ensure that there was no movement of the cells as this would prevent their adhesion to the channel surface. 60 *μ*l of growth media was added to the inlet and the outlet ports of the channel after which the cells were incubated at 37 °C and 5% CO_2_ for 2 h. After 2 h, HUVECs formed a uniform monolayer on the channels and were now ready for perfusion.

### C. Shear stress and pressure distributions

Syringe pumps (World Precision Instruments, Hitchin, UK) were used to achieve the desired shear stress (SS) for different conditions by adjusting the flow rate (*Q*). For a rectangular channel with width 2 W and height 2*H*, the velocity field can be written as^92^

$$u(x,y)=-\frac{16c_{1}W^{2}}{\pi^{3}}\;\sum_{n=0}^{\infty }\frac{{\left(-1\right)}^{n}}{{\left(2n+1\right)}^{3}}\;\left[1-\frac{\text{cosh}\left(\frac{(2n+1)\pi y}{2W}\right)}{\text{cosh}\left(\frac{(2n+1)\pi H}{2W}\right)}\right]\;\text{cos}\left(\frac{(2n+1)\pi x}{2W}\right),$$ (1)

where *c*_1_ depends on the average velocity, $u_{m}=Q/(2W\times 2H)$, and can be written as

$${\;c}_{1}=-3\frac{u_{m}}{W^{2}}\;\frac{1}{1-\frac{192}{\pi^{5}}\;\frac{W}{H}\;\sum_{n=0}^{\infty }\frac{1}{{\left(2n+1\right)}^{5}}\;\text{tanh}\left(\frac{(2n+1)\pi H}{2W}\right)}.$$ (2)

From this, the wall shear stress (WSS) at the bottom wall of the channel (where the cells are cultured) is written as

$$\mu \frac{\partial u(x,y)}{\partial y}{|}_{y=-H}=-\mu \frac{16c_{1}W^{2}}{\pi^{3}}\;\sum_{n=0}^{\infty }\frac{{\left(-1\right)}^{n}}{{\left(2n+1\right)}^{3}}\left[\frac{\left(2n+1\right)}{2W}\;\frac{\text{sinh}\left(\frac{(2n+1)\pi H}{2W}\right)}{\text{cosh}\left(\frac{(2n+1)\pi H}{2W}\right)}\right]\;\text{cos}\left(\frac{(2n+1)\pi x}{2W}\right).$$ (3)

**FIG. 5.**
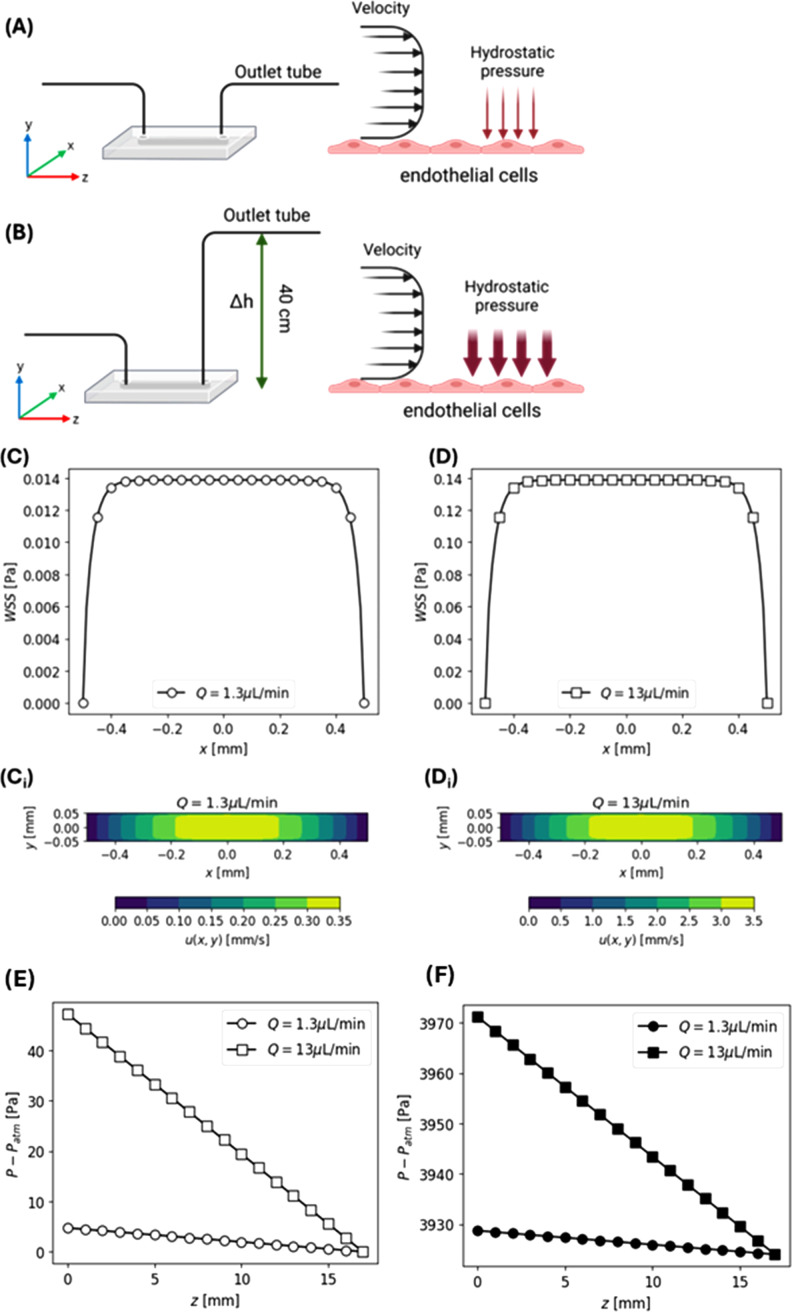
Wall shear stress (WSS) and pressure distribution within the channel. (A) Schematic of the microfluidic channel perfusion during normal flow conditions and (A) during application of elevated hydrostatic pressure conditions wherein the height of the outlet tube was elevated by 40 cm relative to the inlet tube, thereby maintaining the same shear stress profile but increasing the pressure experienced by the cells (*x*, *y*, *z* = *W*, *H*, *L*). (C) and (D) WSS distribution at the channel bottom surface along the *x*-axis derived from the velocity counter plots (C_i_) and (D_i_) for the low flow rate and high flow rate respectively. (E) Pressure distribution along the channel length (*z*-axis) for the low and high flow rates under normal conditions and (F) at elevated hydrostatic pressure conditions.

Figures 5(A) and 5(B) represent the schematic of the microfluidic channel setup during normal and elevated pressure flow conditions, respectively. The “xyz” axis is defined as follow: *x* axis = channel width (*W*) (1 mm), *y* axis = channel height (*H*) (100 *μ*m), and *z* axis = channel length (*L*) (17 mm). Using Eq. (3), the WSS distribution along the bottom wall of the channel (*x* axis) and the corresponding velocity counter plots across the channel cross section (*x-y* axis) for both flow rates, namely, 1.3 *μ*L/min for low shear stress and 13 *μ*L/min for high shear stress experiments, were derived analytically and are depicted in Figs. 5(C) and 5(C_i_) and 5(D) and 5(D_i_), respectively. Both conditions demonstrated a uniform shear stress profile with a peak WSS value of 0.014 and 0.14 Pa for the low and high flow rates, respectively.

Elevated hydrostatic pressure conditions were achieved by raising the outlet tube to 40 cm above the inlet, as shown in Fig. 5(B). At this height, the pressure drop at the channel outlet ( $P_{\text{out}}$) can be derived from

$$P_{\text{out}}=P_{\text{atm}}+\rho gh+\frac{\mu QL}{32\pi D^{4}},$$ (4)

where $P_{\text{atm}}$ is the atmospheric pressure, ρ and μ are the density (1000 kg m^−3^) and dynamic viscosity (0.000 72 Pa s at 37 °C) of the growth medium, and *h* is the height of the outlet tubing (40 cm). Pressure drop due to friction is represented by $\frac{\mu QL}{32\pi D^{4}}$ where in *Q* is the flow rate, *L* is the length of the channel, and *D* is the inner diameter (1 mm) of the outlet tubing. Given the very low flow rates, the friction pressure drop is negligible (<1 Pa) when compared to the atmospheric pressure. Hence the pressure drop at the outlet is $P_{\text{out}}-P_{\text{atm}}=3924\;\text{Pa}$. Since venous blood vessels are the first blood vessels to be impacted by conditions involving elevated compartmental pressure,^93–95^ (due to their low pressure), we wanted to investigate the impact of this elevated pressure (∼4000 Pa) on the endothelial cells of the venules.

For a rectangular channel, the pressure drop along the channel (*z*-axis) can be expressed as^92,96^

$$\frac{dp}{dz}=\frac{\lambda \rho u_{m}^{2}}{2D_{h}},$$ (5)

where $D_{h}$ is the hydraulic diameter and *λ* depends on the aspect ratio of the channel ( α=W/H) and the Reynolds number $\left(Re=\rho u_{m}D_{h}/\mu \right)$,

$$\lambda =\frac{96}{R_{e}}\;(1-1.3553\alpha^{-1}+1.9467\alpha^{-2}-1.7012\alpha^{-3}+0.9564\alpha^{-4}-0.2537\alpha^{-5}).$$ (6)

The pressure at a given point in the channel, with *P_out_* being the pressure at the exit of the channel, *L* being the channel length, and *z* being the position from the inlet (i.e., *L*–*z* is the position in the channel relative to the outlet) is given by

$$p(z)=P_{\text{out}}+(L-z)*\frac{dp}{dz}.$$ (7)

The $P_{\text{out}}-P_{\text{atm}}$ at inlet *p* (*z* = 0) for SS low only and SS high only is 4.71 and 47.1 Pa, respectively, and the $P_{\text{out}}-P_{\text{atm}}$ at the outlet *p* (*z* = 17) is 0 for both conditions as shown in Fig. 5(E). Hence, the pressure difference (ΔP) along the channel for SS low only and SS high only is 4.71 and 47.1 Pa respectively. Similarly in the HP conditions, $P_{\text{out}}-P_{\text{atm}}$ at the inlet *p* (*z* = 0) for SS low+HP and SS high+HP is 3928.71 and 3971.18 Pa, respectively, and at the outlet *p* (*z* = 17), $P_{\text{out}}-P_{\text{atm}}$ is 3924 Pa for both conditions as shown in Fig. 5(F). Hence, SS low+HP and SS high+HP demonstrate a similar ΔP of 4.71* and* 47.1 Pa, respectively, along the channel. The full range of the $P_{\text{out}}-P_{\text{atm}}$ values at every point of p(z) for both flow rates (±HP) has been provided in the supplementary material Sec. 1.3 (via GitHub link).

A summary of the two flow rates used, shear stress and pressure at the inlet, is presented in Table I.

### D. Mechanosensitive channels inhibition

For mechanosensitive channels inhibition experiments, cells were exposed to growth media that contained GsMTx4 (Tocris bioscience, Bio-Techne, UK) which is a peptide derived from tarantula venom that selectively inhibits mechanosensitive cationic channels, including TRPC1, TRPC6, and piezo-1.^53,97^ GsMTx4 was dissolved in growth media to give a final working solution of 5 *μ*M concentration. After exposure, cells were washed thrice with DPBS and were fixed for immunostaining.

### E. Immunofluorescence staining

Immunofluorescence staining was performed by flowing reagents on top of the cells within the microfluidic devices. For immunofluorescent staining, the cells were fixed by flowing 10% Formalin neutral buffered solution (Sigma Aldrich, Merck Ltd, Dorset, UK) for 10 min at room temperature into the microchannel followed by incubation with 0.1% Triton-X in Dulbecco's phosphate buffered saline (DPBS, Gibco, Merck Ltd, Dorset, UK) for 5 min at room temperature for permeabilization of the cell membrane. The channels were then washed 3 times using DPBS and were subsequently blocked using 1% BSA (Sigma Aldrich, Merck Ltd, Dorset, UK) diluted in DPBS containing 0.1% Tween-20 (Sigma Aldrich, Merck Ltd, Dorset, UK) for 30 min. The channels were washed thrice with DPBS and were incubated with 5 *μ*g/ml (diluted in blocking buffer) mouse anti-human monoclonal VE-cadherin (CD144) primary antibodies (Invitrogen, Fisher Scientific, Loughborough, UK) at 4 °C overnight. The following day, the unbound primary antibodies were removed by washing the channels three times with DPBS and the cells were subsequently incubated for 1 h at room temperature with goat anti-mouse secondary antibodies conjugated with Alexa Fluor™ 488 (Invitrogen, Fisher Scientific, Loughborough, UK) at a dilution of 1:1000 in blocking buffer. After 1 h, the unbound secondary antibodies were removed by washing 3 times with DPBS. The cells were then stained for F-actin using Phalloidin CF^®^594 (Biotium, Inc., CA, USA) as well as nuclei using DAPI (4′,6-diamidino-2-phenylindole, Sigma Aldrich, Merck Ltd, Dorset, UK) for 5 min at room temperature. The cells were then imaged (while still within the microfluidic device) using a Carl Zeiss LSM880 scanning confocal microscope predominantly using the 40× (C-Apochromat, 1.2 W Korr FCS M27) and 63× (C-Apochromat, 1.2 W Korr M27) objectives.

### F. Image analysis

All the raw images were processed (brightness/contrast adjustments) either using FIJI (US National Institutes of Health) or Zeiss Zen software. Cells were predominantly imaged around the middle area of the channel cross section where peak WSS was experienced. Cells at the very edges of the channel (growing on the side walls) and cells around the inlet/outlet ports were avoided. For VE-cadherin and actin co-localization plot profiles on FIJI, a straight line was drawn across a cell-cell junction. For all other cell morphological analyses, customized pipelines on CellProfiler 4.2.6 (Ref. 98) (Broad Institute, Massachusetts Institute of Technology, USA) were used to automate image analysis. Briefly, nuclei and actin raw images, designated as primary and secondary objects, respectively, were thresholded using a minimum cross-entropy method. Cytoplasm fluorescence intensity was derived by subtracting the primary object (DAPI stain) from the secondary object (whole cell stain, which in this case is actin stain). Cell area, circularity, compactness, and whole cell VE-cadherin fluorescence intensity were quantified using the “Measure ObjectSizeshape” and “Measure ObjectIntensity” modules. Membrane VE-cadherin fluorescence intensity was measured using a modified pipeline. Briefly, the primary and secondary objects were obtained using the DAPI and actin images. The secondary object was then shrunk by 3 pixels using the “ExpandorShrinkObjects” module. The cell membrane of 3 pixel thickness was isolated by subtracting the shrunk secondary object from the whole secondary object and the VE-cadherin fluorescence intensity was measured using the “Measure ObjectIntensity” module. The pipelines used for image analysis on CellProfiler can be provided upon request.

### G. Statistics

All statistical analysis was performed using GraphPad Prism10.3.1. One-way ANOVA along with Tukey's post-hoc test was used to compare between different conditions. Statistical significance was set as follows: ^*^p < 0.05, ^**^p < 0.01, ^***^p < 0.001, and ^****^p < 0.0001. Statistical tests and relative p values are indicated in each figure legend. Unless stated elsewhere, all experiments were performed with at least three biological independent (three channels) replicates.

## SUPPLEMENTARY MATERIAL

See the supplementary material for information on (1) actin cytoskeletal alignment at 1 h elevated hydrostatic pressure condition, (2) comparison of VE-cadherin finger formation at high shear stresses, (3) actin-VE-cadherin colocalization in the 24 h prolonged elevated hydrostatic pressure exposure, and (4) analytically derived pressure distribution at every point p(*z*) along the channel length for both shear stress conditions.

## Data Availability

The data that support the findings of this study are available within the article and its supplementary material. Further data that support the findings of this study are available from the corresponding authors upon reasonable request
